# Engineering Plant-Associated Microorganisms for Bioremediation and Sustainable Agriculture

**DOI:** 10.3390/microorganisms14061203

**Published:** 2026-05-26

**Authors:** Aurora I. Flores, Luzmaría R. Morales-Cedeño, Pedro D. Loeza-Lara, Mauricio Schoebitz, Ma. del Carmen Orozco-Mosqueda, Gustavo Santoyo

**Affiliations:** 1 Genomic Diversity Laboratory, Institute of Biological and Chemical Research, Universidad Michoacana de San Nicolas de Hidalgo, Morelia 58030, Mexico; aurora.flores@umich.mx (A.I.F.); luzmaria.morales@umich.mx (L.R.M.-C.); 2 Food Genomics, Universidad de La Ciénega del Estado de Michoacán de Ocampo, Sahuayo 59103, Michoacán, Mexico; pdloeza@ucemich.edu.mx; 3 Department of Soil Science and Natural Resources, Faculty of Agronomy, Universidad de Concepción, Concepción P.O. Box 160 C, Chile; mschoebitz@udec.cl; 4 Center of Biotechnology, Universidad de Concepción, Concepción P.O. Box 160 C, Chile; 5 Department of Biochemical and Environmental Engineering, National Technological Institute of Mexico in Celaya, Celaya 38010, Guanajuato, Mexico; carmen.orozco@itcelaya.edu.mx

**Keywords:** bioremediation, CRISPR, genome modification, rhizosphere

## Abstract

As food demand increases, agricultural practices have evolved, prompting increased exploration of sustainable ecological techniques and utilization of plant-associated microorganisms. In this context, plant fitness has been enhanced by plant growth-promoting microorganisms (PGPM), which stimulate growth through direct mechanisms, such as improved nutrient availability and phytohormone production, as well as indirect mechanisms, including protection against phytopathogens and suppression of soil-borne diseases. However, these innate capabilities of PGPM can be further improved through genomic modification or editing. This article reviews advances in the genomic engineering of plant-beneficial microorganisms as tools to enhance their positive effects on crop performance and environmental remediation. The genetic modification strategies analyzed here include random mutagenesis, targeted genome editing (such as CRISPR-Cas), gene over-expression, genome shuffling, RNA interference, metabolic pathway engineering, and synthetic biology approaches. These tools have enabled the optimization of functions, such as nitrogen fixation, phosphate solubilization, secondary metabolite production, biocontrol, stress tolerance, and bioremediation. However, we propose expanding the discussion of their regulation and use in various countries. Additionally, these modifications must be efficient and safe for the beneficial microbiota associated with the target crop, as well as for humans, animals, and the environment, all of which depend on sustainable agricultural practices.

## 1. Introduction

Global population growth continues to be a major concern, with projections from the United Nations Department of Economic and Social Affairs estimating that the world population will reach 8.5 billion by 2030 and 9.7 billion by 2050 [1]. This demographic expansion is expected to significantly increase food demand, which may surpass agricultural production under current farming practices [2]. To address this challenge, modern agriculture has heavily relied on the widespread use of chemical fertilizers to enhance crop yield [3]. While effective in boosting productivity, their excessive and prolonged use has led to severe environmental consequences, including soil acidification, nitrogen leaching into groundwater, eutrophication of aquatic ecosystems, soil degradation, biodiversity loss, and increased greenhouse gas emissions [4,5]. Moreover, exposure to chemical fertilizers has been associated with adverse human health effects, particularly affecting liver and kidney function [6].

In parallel, crop productivity is further constrained by multiple biotic and abiotic stress factors, including plant pathogens, insect and arthropod predation [7], climate change [7,8], salinity [9], environmental pollution [10], soil degradation [11], and other stress conditions [12]. These challenges highlight the urgent need for sustainable agricultural strategies that can maintain high productivity while preserving environmental integrity and plant health. In this regard, the development and implementation of integrated approaches that simultaneously enhance crop yield, resilience, and ecosystem stability have become global priorities [13,14,15].

Thus, plant growth-promoting microorganisms (PGPM) have emerged as an alternative to agrochemicals in the pursuit of more sustainable agriculture. PGPM enhance plant growth through direct mechanisms, including phytostimulation—mediated by the production of phytohormones such as indole-3-acetic acid (IAA), cytokinins, and gibberellins, as well as modulation of ethylene levels via ACC deaminase—and biofertilization processes such as biological nitrogen fixation, phosphate solubilization (e.g., via gluconic, citric, and oxalic acids), potassium mobilization, and siderophore-mediated iron acquisition. Representative plant-beneficial rhizobacteria include species within the genera *Pseudomonas*, *Bacillus*, as well as rhizobia such as *Rhizobium* and *Bradyrhizobium*, which are well known for their multifunctional roles in nutrient cycling and plant stimulation. In addition, PGPM improve plant performance through indirect mechanisms, including biocontrol—via the production of antimicrobial compounds (e.g., lipopeptides such as surfactin, iturin, and fengycin), antibiotics, volatile organic compounds (VOCs), and lytic enzymes (e.g., chitinases, glucanases, and proteases)—as well as rhizoremediation and stress mitigation through osmolyte production, antioxidant enzyme induction, and systemic resistance activation. These beneficial traits are also widely documented in filamentous fungi such as *Trichoderma* spp., grass-associated endophytes such as *Epichloë* spp., and root endophytes such as *Serendipita indica* [16,17].

In this context, microalgae are increasingly recognized as complementary PGPM due to their capacity to modulate plant development through the production of phytohormones, release of bioactive metabolites, and contribution to nutrient mobilization and rhizosphere stabilization. Among them, *Chlamydomonas reinhardtii* stands out as a model species capable of producing indole-3-acetic acid and other signaling molecules that influence root development. Its well-characterized genome and advanced genetic toolkit enable precise metabolic engineering, positioning it as a promising chassis for the development of next-generation bioinoculants with enhanced functional performance under agricultural conditions [18].

However, despite the well-documented benefits of plant growth-promoting microorganisms (PGPM), their translation from controlled conditions to field applications remains inconsistent. This limitation largely arises from environmental variability, competition with resident microbiota, and complex rhizosphere interactions that constrain stable colonization and functional expression of beneficial traits. Model genera such as *Pseudomonas* and *Bacillus*, although extensively characterized for their plant-beneficial activities, often display context-dependent performance, highlighting the need for strategies that enhance their ecological fitness and functional reliability [19].

Advances in genetic engineering provide a powerful framework to address these constraints by enabling the rational improvement of microbial traits. According to the World Health Organization (WHO), genetically modified organisms (GMOs) are those whose genetic material has been altered using modern biotechnological tools. In microorganisms, these approaches include precise genome editing (e.g., CRISPR-Cas systems), gene over-expression, pathway rewiring, and synthetic biology-driven circuit design, allowing the enhancement of key functions such as root colonization, stress resilience, and the biosynthesis of phytohormones and antimicrobial metabolites [17].

While most commercial GMOs to date correspond to engineered crops with resistance to biotic or chemical stresses [20], increasing attention is being directed toward genetically engineered microorganisms (GEMs) as next-generation agricultural inputs. In this context, bacterial systems such as *Pseudomonas* and *Bacillus*, along with fungal platforms such as *Trichoderma*, represent highly tractable models for engineering multifunctional traits, including enhanced biocontrol activity, nutrient mobilization, and environmental adaptability. In addition to promoting plant growth, GEMs offer significant potential for rhizosphere engineering and bioremediation through their targeted metabolic capabilities.

**Figure 1:**
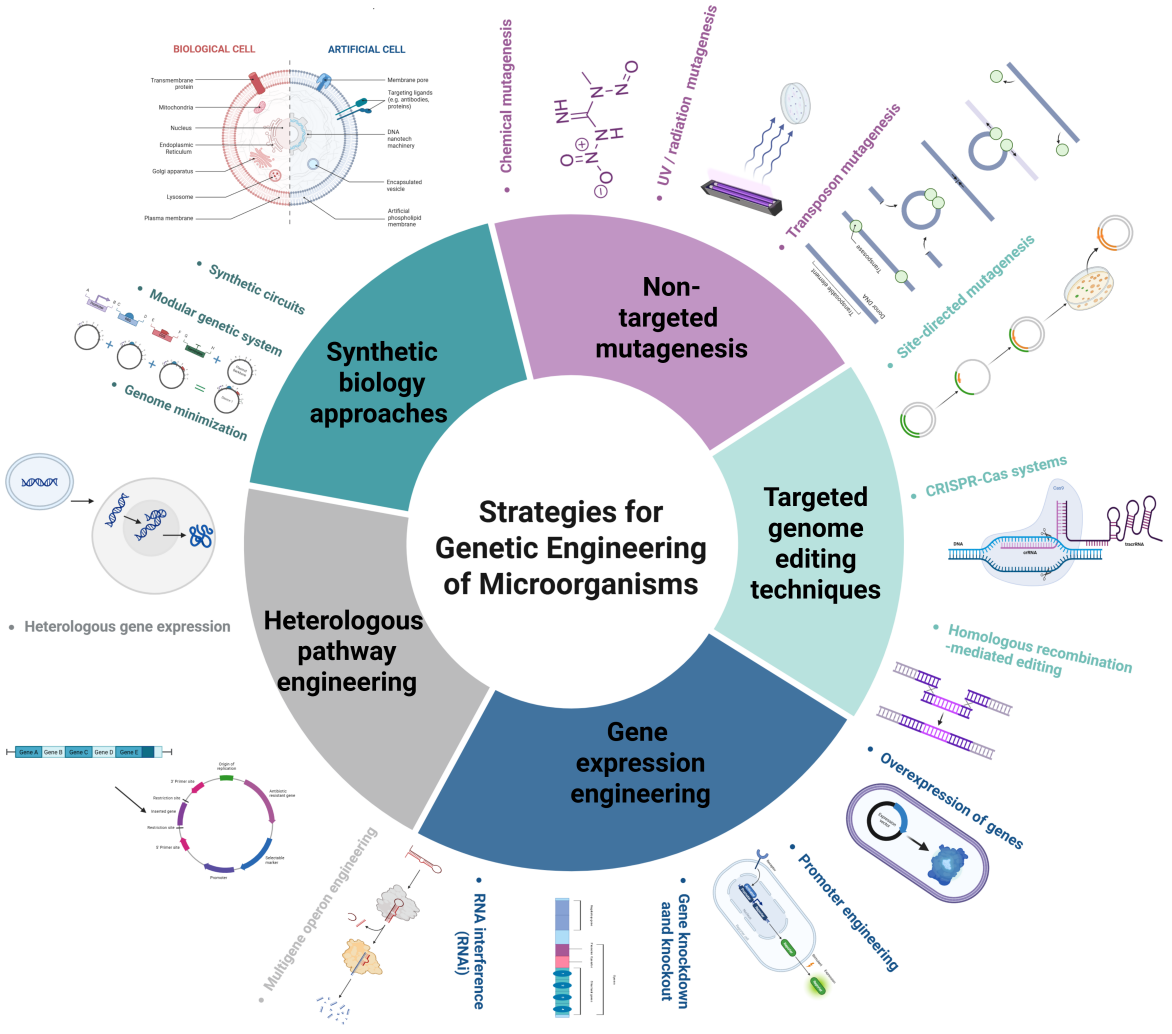
Strategies for Genetic Engineering of Microorganism.

Therefore, this review provides a comprehensive overview of current genetic/genomic engineering strategies applied to microorganisms with agricultural relevance (Figure 1). We discuss key technological advances, their application to model PGPM systems, and the opportunities and limitations associated with their deployment as bioinoculants, biocontrol agents, and bioremediators in sustainable agricultural systems.

The diagram summarizes the five main approaches discussed in this review: (1) non-targeted mutagenesis, including chemical, physical (radiation), and transposon-based methods; (2) targeted genome editing techniques, such as site-directed mutagenesis, CRISPR–Cas systems, and homologous recombination; (3) gene expression engineering, including gene over-expression and RNA interference; (4) heterologous pathway engineering for the introduction and optimization of biosynthetic pathways in alternative hosts; and (5) synthetic biology approaches based on the design and construction of modular genetic systems. Created in https://BioRender.com.

## 2. Biocontrol and Plant Growth Promotion Strategies

Biological approaches to crop protection and productivity enhancement have gained increasing attention as sustainable alternatives to conventional agricultural practices in recent years. This topic has been extensively reviewed by leading experts in the field [21]; therefore, we provide only a brief overview of the general mechanisms underlying biocontrol and plant growth promotion. In this context, a biocontrol mechanism can be defined as any process in which the survival or activity of a pathogen is reduced through the action of other organisms, ultimately leading to a decrease in disease incidence [22]. Recently, microbiological tools used in crop cultivation have predominantly focused on bacteria, yeasts, and soil-associated fungi, with comparatively less attention given to other microbial groups with potential agricultural relevance [23,24,25].

According to Pirttilä et al. [25], improving plant health presents several challenges, including the continuous search for efficient microorganisms capable of supporting plant growth under diverse stress conditions. Likewise, microorganisms face multiple limitations that can affect their performance in the field, such as difficulties in monitoring, environmental sensitivity, transient persistence in soil, limited host range, competition with native microbiota, and constraints related to formulation stability and industrial scalability [23]. Furthermore, a comprehensive evaluation of microorganisms used as bioinoculants and biofertilizers is essential, moving beyond laboratory and in vitro studies, as agricultural conditions within the plant holobiont are significantly more complex and dynamic [26].

In recent years, omics-based approaches have been increasingly used to study plant growth promotion and biocontrol of phytopathogens, enabling a deeper understanding of plant–microbe and microbe–microbe interactions at multiple biological levels. Comparative genomics, for instance, has been used to identify gene clusters associated with antimicrobial compound production and rhizosphere competence, as shown in *Pseudomonas* and *Bacillus* species [27]. Likewise, transcriptomic analyses have revealed key regulatory networks involved in root colonization, such as the expression of adhesion factors, motility-related genes, and biofilm formation in *Bacillus subtilis* and *Pseudomonas fluorescens* [28]. Functional genomics studies have further identified critical genes required for efficient root colonization, including those encoding flagellar components, exopolysaccharides, and quorum sensing systems [29].

Metabolomics has also provided valuable insights into the biosynthesis of secondary metabolites, such as lipopeptides (e.g., surfactin, iturin, and fengycin in *Bacillus* spp.) and phenazines or pyoluteorin in *Pseudomonas* spp., which are directly involved in pathogen suppression [30]. In parallel, omics approaches have elucidated mechanisms underlying tolerance to abiotic stresses and nutrient acquisition, including osmoprotectant synthesis, ion transport regulation, and enhanced nitrogen and phosphorus metabolism [31]. Additionally, studies have demonstrated how beneficial microorganisms activate plant defense systems, for example, through induced systemic resistance (ISR) mediated by jasmonic acid and ethylene signaling pathways [32].

At the community level, recent research has shown that specific bioinoculants can shape the rhizosphere microbiome by recruiting beneficial microbial consortia, as observed in crops such as maize and wheat, where enrichment of *Actinobacteria* and *Proteobacteria* is associated with improved plant health and disease suppression [33]. Furthermore, plant responses to microbial volatiles—such as 2,3-butanediol and acetoin produced by *Bacillus* spp.—have been linked to growth promotion and systemic resistance [34]. These advances have driven the development of genetically engineered microorganisms (GEMs) with enhanced biocontrol and plant growth-promoting capabilities [35]. Integrated multi-omics analyses have also uncovered complex metabolic and transcriptional reprogramming underlying plant resistance to pathogens, including coordinated regulation of defense-related genes, phytohormone signaling, and secondary metabolism [36].

Consequently, genetic modification strategies for PGPM have focused on enhancing key functional traits. These include improved nutrient acquisition, such as biological nitrogen fixation through the optimization of *nif* gene clusters in *Azospirillum* and *Rhizobium* [37,38,39,40], and phosphate solubilization via over-expression of organic acid production pathways in *Pseudomonas* and *Bacillus* species solubilization [40,41,42,43,44,45]. Other strategies involve increasing siderophore production (e.g., pyoverdine in *Pseudomonas*) [46,47,48], boosting the synthesis of antimicrobial secondary metabolites [49,50,51,52,53], and enhancing the expression of hydrolytic enzymes such as chitinases, glucanases, and proteases that degrade fungal cell walls [54,55,56].

Additional targets include improving root colonization through the regulation of chemotaxis, motility, and biofilm formation, as well as strengthening biocontrol traits by engineering pathways involved in antibiotic production and competition with pathogens [39,40,57]. Moreover, efforts to increase stress tolerance have focused on traits such as ACC deaminase activity, osmolyte production (e.g., proline, trehalose), and reactive oxygen species (ROS) detoxification mechanisms [42,47,58,59].

**Figure 2:**
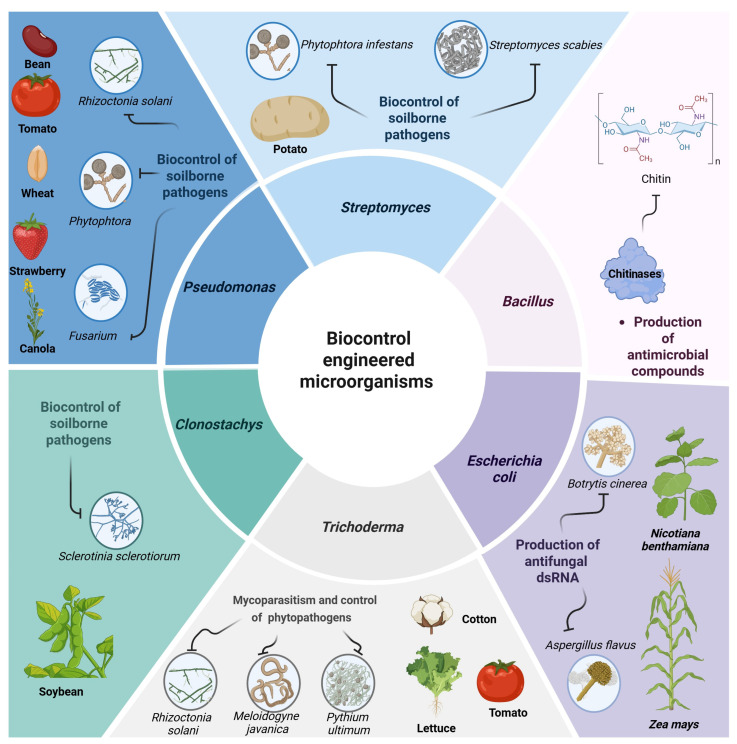
Overview of biocontrol engineered microorganisms and their associated mechanisms. The diagram highlights representative genera featured in this review, such as *Streptomyces*, *Bacillus*, *Escherichia coli*, *Trichoderma*, *Clonostachys*, and *Pseudomonas*. Created in https://BioRender.com.

Recent advances in omics approaches have led to a surge in studies linking microbial genes to plant phenotypes under field conditions [60]. While these correlation-based analyses provide valuable insights into community structure and potential functions, they do not establish causality. Distinguishing correlation from causation remains a key challenge in the study of plant growth-promoting microorganisms (PGPM). Functional validation—through gene manipulation, mutant analysis, and controlled or semi-controlled inoculation assays—is essential to confirm that inferred mechanisms directly contribute to plant growth promotion or biocontrol. Without such validation, associative data risk being overinterpreted, particularly under variable field conditions. Integrating omics with rigorous experimental validation is therefore critical to achieve mechanistically grounded and reliable agricultural applications, supporting a shift from conventional inoculant selection toward microbiome engineering strategies [61]; importantly, genetically engineered microorganisms are also integral to these validation frameworks, enabling direct testing of causal relationships between specific traits and plant responses (Figure 2).

## 3. Genomic Engineering Strategies in Microorganisms

### 3.1. Non-Targeted Mutagenesis

A mutant arises from spontaneous or experimentally induced mutagenesis. The effects of mutations can be classified as direct or indirect. Direct effects involve genomic alterations that modify gene structure or expression, whereas indirect effects impact the organism’s phenotype. Silent mutations consist of nucleotide changes that do not affect gene function, typically occurring in intergenic or non-coding regions. In contrast, mutations in coding regions are particularly relevant, as they may lead to functional changes in gene products [62].

Non-targeted mutagenesis, also known as random mutagenesis, is induced by chemical or physical mutagens, generating mutations across the genome without predefined targets. Mutation frequencies vary widely depending on the mutagen and organism, typically ranging from ~10^−6^ to 10^−3^ mutations per gene per generation, often requiring the screening of large mutant populations to identify desirable phenotypes. Although this approach has been widely used in breeding programs, its applications are limited by its unspecific nature, low efficiency in recovering beneficial mutations, and the time-consuming processes required for mutant selection and characterization [63]. In contrast to modern genome-editing technologies such as CRISPR-based systems, which enable precise, targeted, and often predictable modifications at specific loci, non-targeted mutagenesis lacks control over mutation location and outcome [64]. Nevertheless, mutations—whether spontaneous or induced—remain a valuable source of genetic diversity. They may involve single base changes (e.g., substitutions, inversions, or deletions) or larger-scale events such as gene rearrangements, chromosomal alterations, or insertion of mobile elements that affect gene structure and expression. Mutations arise through two main mechanisms: spontaneous mutations result from errors during DNA replication, whereas induced mutations are caused by physical or chemical mutagens, or biological agents such as viruses [62]. In contrast, induced mutations are caused by physical or chemical mutagens, or biological agents such as viruses, which disrupt normal base pairing and can also be inherited. Despite being relatively imprecise, mutagenesis remains widely used due to its simplicity and capacity to generate broad phenotypic diversity [65].

#### 3.1.1. Chemical Mutagenesis

Chemical mutagenesis is a classical strategy for generating mutants, valued for its simplicity, flexibility, and low cost, as it does not require exogenous DNA [66]. Common mutagens include diethyl sulfate (DES), ethyl methanesulfonate (EMS), isopropyl methanesulfonate (iPMS), ethylenimine (EI), N-nitroso-N-ethylurea (NEU), N-nitroso-N-methylurea (NMU), 1,4-bisdiazoacetylbutane, sodium azide (NaN_3_), and base analogs such as 5-bromodeoxyuridine and 2-aminopurine [65,67,68]. These agents primarily induce mutations through alkylation, base modification, or mispairing, leading to nucleotide substitutions [65]. Despite its widespread use, chemical mutagenesis has important limitations. The process is random and non-targeted, often resulting in deleterious effects, metabolic instability, or loss of beneficial traits. Consequently, extensive screening is required to identify desirable phenotypes, making it labor-intensive. In addition, the hazardous nature of many mutagens and the lack of precision have driven the adoption of safer and more controllable genome engineering approaches in modern microbial biotechnology.

#### 3.1.2. UV/Radiation Mutagenesis

The mutagenic effects of radiation were proposed in the early 20th century and later confirmed by Hermann J. Muller in fruit flies [69] and Lewis J. Stadler in barley [70]. Common physical mutagens include ultraviolet (UV) radiation, X-rays, gamma rays, cosmic rays, and particle radiation (e.g., α and β particles, neutrons), as well as emerging approaches such as atmospheric and room-temperature plasma (ARTP) and laser radiation [65,68]. UV radiation remains a classic tool for random mutagenesis, primarily inducing cyclobutane pyrimidine dimers and 6–4 photoproducts that block DNA replication [71]. However, its effectiveness may be reduced in phototrophic organisms due to photoprotective pigments and efficient DNA repair systems. In contrast, ionizing radiation (e.g., X-rays and gamma rays) induces more severe damage, including deletions, chromosomal fragmentation, and translocations, thereby increasing mutation rates and generating broader phenotypic diversity. These genomic alterations have been exploited to obtain plant-associated microorganisms with improved functional traits, such as enhanced production of phytohormones, antibiotics, and hydrolytic enzymes, as well as increased tolerance to abiotic stresses. Emerging technologies such as ARTP and laser-based mutagenesis offer advantages such as short exposure times and high mutant diversity, facilitating the identification of variants with improved plant growth-promoting or biocontrol capacities [72]. Nevertheless, physical mutagenesis requires specialized infrastructure and often lacks precise control, with limited data on mutation rates, cell survival, and long-term genetic stability of induced mutations [65].

#### 3.1.3. Transposon Mutagenesis

Transposons, or transposable elements (TEs), are mobile DNA sequences widely distributed across genomes [73,74]. They are present in all domains of life and contribute to processes such as adaptation, gene regulation, and the generation of genetic diversity [75]. TEs are broadly classified into two types: retrotransposons and DNA transposons, both of which have been adapted as tools for genome engineering [74,76,77].

Due to their ability to mediate stable DNA insertions, transposons are powerful tools in functional genomics. They can carry relatively large DNA fragments or multiple genes and are generally more cost-effective than viral vectors [73]. However, their semi-random integration can disrupt essential genes, potentially causing genomic instability and unintended mutational effects [74].

Among the most widely used transposon systems are Sleeping Beauty (SB), piggyBac (PB), and Tol2, which have been successfully applied for exogenous DNA insertion across diverse organisms, from cultured cells to vertebrates [73]. In bacteria, one of the most extensively studied systems is Tn5, a DNA transposon that operates via a “cut-and-paste” mechanism mediated by a transposase that recognizes inverted repeat sequences. Beyond its mechanistic relevance, Tn5 has been widely used in plant growth-promoting microorganisms (PGPM) to generate insertional mutant libraries for identifying genes involved in key traits such as phytohormone production, nutrient solubilization, root colonization, and biocontrol activity. By linking gene disruption to phenotypic outcomes, Tn5-based approaches enable the functional characterization of PGPM. While similar insertional mutagenesis strategies have been explored in other systems, including microalgae, their application remains comparatively limited and less standardized. These aspects are discussed in more detail in subsequent sections [77].

### 3.2. Targeted Genome Editing

#### 3.2.1. Site-Directed Mutagenesis

Targeted mutagenesis is a fundamental tool in modern genetic engineering, enabling precise and controlled modifications of defined DNA sequences. Unlike random mutagenesis, it facilitates the rational evaluation and optimization of specific phenotypic traits [78].

Among these approaches, site-directed mutagenesis (SDM) stands out for its ability to introduce specific nucleotide changes, including multiple mutations in a single reaction. The method relies on the design of oligonucleotide primers carrying the desired modifications, which are incorporated into plasmid DNA through PCR, generating precise sequence alterations [79].

In addition to point mutations, SDM allows for insertions and deletions. However, its efficiency can be reduced in GC-rich regions or sequences with repeats and strong secondary structures, which hinder primer annealing and PCR amplification. Moreover, successful implementation requires prior knowledge of the target sequence [78,79,80].

#### 3.2.2. CRISPR-Cas Systems

CRISPR–Cas systems have become the leading genome-editing technology. Originally identified as an adaptive immune mechanism in bacteria and archaea, they protect against bacteriophages and foreign DNA by incorporating fragments of invading sequences into CRISPR arrays, which are later recognized and targeted for degradation [81].

Genome editing with CRISPR–Cas relies on a guide RNA (gRNA) that directs a Cas nuclease to a specific DNA sequence adjacent to a protospacer adjacent motif (PAM). The nuclease introduces a double-strand break, which is repaired by cellular mechanisms. Two main repair pathways are involved: non-homologous end joining (NHEJ), which typically generates insertions or deletions, and homology-directed repair (HDR), which enables precise sequence modifications using a DNA template [82,83].

CRISPR–Cas systems are classified into two major classes. Class 1 (types I and III) employs multi-protein effector complexes and is predominantly found in archaea, whereas Class 2 (types II, IV, V, and VI) relies on a single Cas nuclease and is more common in bacteria [84,85].

Compared with earlier genome-editing tools, such as meganucleases, zinc finger nucleases (ZFNs), and TAL effector nucleases (TALENs), CRISPR–Cas systems are simpler and more versatile, as retargeting only requires redesigning the gRNA [82]. Beyond generating gene knockouts or insertions, CRISPR technologies enable gene regulation, epigenetic editing, and high-throughput functional screening [82,83].

These features have positioned CRISPR–Cas as a powerful platform across multiple fields. In medicine, it has shown promise in correcting genetic mutations and treating monogenic disorders such as sickle cell anemia and other hemoglobinopathies, as well as in cancer and infectious disease research [82,86,87,88,89,90]. In agriculture, CRISPR-based approaches have transformed crop improvement by enabling precise modifications associated with stress tolerance and productivity [91,92,93,94,95].

#### 3.2.3. Homologous Recombination/Recombineering

Homologous recombination is a molecular process in which DNA molecules with identical or highly similar sequences exchange genetic information. It plays a central role in DNA repair, particularly in the correction of double-strand breaks and the maintenance of genomic stability [96].

In genetic engineering, this mechanism is exploited to achieve targeted genome modification. Exogenous DNA is designed with homologous flanking regions that direct its integration into a specific genomic locus. Sequence exchange occurs only when sufficient homology exists between the donor DNA and the target site, with the required length depending on the recombination system used [97,98].

Several recombineering strategies have been developed based on this principle, including RecA-dependent recombination, the λ Red and RecET systems, and integration via suicide plasmids [97]. In eukaryotes, gene targeting approaches and homology-directed repair (HDR) are widely used [99]. HDR can be enhanced by programmable nucleases, such as CRISPR–Cas, TALENs, and ZFNs, which introduce site-specific double-strand breaks, facilitating precise genetic modifications [82,100].

### 3.3. Gene Expression Engineering

#### 3.3.1. Gene Over-Expression

Gene over-expression involves increasing the expression of a gene beyond its native levels or regulatory patterns, most commonly in unicellular systems [101]. This strategy underpins the heterologous production of recombinant proteins and has been widely used for the large-scale synthesis of biotechnologically relevant compounds.

Successful implementation requires careful consideration of several factors, including the selection of an appropriate host organism (e.g., bacteria, yeasts, fungi, or microalgae), the design of a suitable expression vector, promoter choice, selectable markers, and the incorporation of affinity tags to facilitate protein purification. Despite its advantages, gene over-expression can impose a metabolic burden on the host. The production of non-essential or excessive proteins may disrupt cellular homeostasis, impair growth, or even result in toxicity, limiting the efficiency of recombinant protein expression systems [102].

#### 3.3.2. RNA Interference (RNAi)

RNA interference (RNAi) is a gene-silencing technology widely used across biological systems, particularly in plant defense [103]. It operates by introducing double-stranded RNA (dsRNA) into cells, triggering the degradation or suppression of target RNA molecules in a sequence-specific manner [104,105]. Recent advances include the development of small artificial RNAs, which enable more precise and efficient gene regulation.

RNAi has also gained importance in environmental and agricultural applications as an eco-friendly strategy for pest and disease control. Its high specificity allows targeted action while minimizing off-target effects on non-target organisms [106]. RNAi approaches can be broadly classified into two types: (1) endogenous expression, where dsRNA is produced within the target organism (either stably or transiently), and (2) exogenous application, in which RNAi molecules are generated externally and subsequently taken up by the organism [104].

This technology has been successfully applied to control a wide range of agricultural pests, including insects (e.g., *Sitobion avenae*, *Schizaphis graminum*, *Lygus lineolaris*, *Acyrthosiphon pisum*, and *Helicoverpa armigera*) [107,108,109,110,111], phytopathogenic fungi (e.g., *Botrytis cinerea*, *Phytophthora parasitica*, and *Fusarium verticillioides*) [111,112,113,114], *Phytophthora parasitica* var. *nicotianae* [115], and plant-parasitic nematodes (e.g., *Heterodera schachtii*, *Meloidogyne javanica*, *Pratylenchus vulnus*, *Meloidogyne enterolobii*, and *Ditylenchus destructor*) [116,117,118,119,120].

### 3.4. Heterologous Gene Expression

The increasing availability of genomic data, together with advances in genome mining and biosynthetic gene cluster (BGC) annotation, has greatly expanded the discovery and optimization of specialized metabolites. These approaches have enabled the identification of silent or poorly expressed BGCs in native hosts, uncovering new sources of bioactive compounds [121].

Heterologous expression has emerged as a key strategy for the functional characterization and production of BGCs in alternative hosts. This can be achieved through multicopy plasmid systems or stable genomic integration, which improves genetic stability and allows tighter control of gene expression [121]. Beyond simple gene transfer, heterologous expression enables the introduction of new metabolic capabilities, activation of cryptic biosynthetic pathways, and reinforcement of specific biochemical routes. As such, it represents a powerful tool for the targeted production of valuable metabolites and the redesign of microbial metabolism [102,121,122,123].

### 3.5. Synthetic Biology Approaches

Synthetic biology (SB) is an engineering-driven discipline focused on the design and construction of biological systems and functions that do not naturally occur [124]. It enables the rational assembly of genes and modular biological components—either naturally derived or synthetically designed—to reprogram and redesign cellular functions [125]. SB integrates multiple fields, including molecular biology, systems biology, chemistry, biophysics, and computational modeling [126].

A central concept in SB is the development of genetic circuits, inspired by electronic logic gates, which allow cells to sense, process, and respond to specific signals. These circuits typically consist of three core elements: sensors, processors, and actuators, enabling programmable control of cellular behavior [127]. Recent advances have expanded the library of standardized and interchangeable biological parts, commonly known as BioBricks. These include synthetic promoters, riboswitches, engineered transcription factors, ribosome binding sites (RBS), coding sequences, and terminators. Among these, synthetic promoters play a key regulatory role, as they can be rationally designed to control transcription through specific interactions with transcription factors and the transcriptional machinery [128]. This modular architecture enhances the ability to design, assemble, and fine-tune biological systems, facilitating the production of enzymes, specialized metabolites, and engineered phenotypes, as well as enabling the generation of detectable biological signals [129].

### 3.6. Synthetic Genomes and Genome Minimization

The design of synthetic and minimal genomes represents a major recent advance in biotechnology and genetic engineering. Early work by Hutchison et al. [75], established a minimal gene set in *Mycoplasma genitalium*, initially comprising 525 genes, which was later reduced to approximately 250 genes [130]. These studies demonstrated that bacterial cells can be sustained with highly reduced, artificially designed genomes. Genome reduction, achieved through the elimination of non-essential sequences, can facilitate the incorporation of heterologous biosynthetic pathways and improve the efficiency of engineered functions. Consequently, genome minimization has emerged as a promising strategy for developing optimized microbial platforms in biotechnology and agriculture.

However, this approach should be applied with caution. Genes considered non-essential under laboratory conditions may play important roles in complex environments, such as the rhizosphere or during endophytic colonization of plants. Given that these interactions are not yet fully understood, the removal of such genes could compromise traits related to plant growth promotion or environmental fitness.

## 4. Genetically Engineered Microorganisms

GEMs are organisms from all domains of life whose genomes have been deliberately modified using genetic engineering tools. These organisms have broad applications across multiple sectors. In the food industry, they are used as ingredients and processing aids [131]; in agriculture, GEMs enhance nutrient availability, improve plant–microbe interactions, and function as biocontrol agents. Additionally, they play important roles in industrial and environmental applications, including the production of biofuels, commodity chemicals, and advanced materials, as well as in bioremediation processes. This review focuses on GEMs developed for agricultural applications, particularly those designed to promote plant growth and sustainability, as well as microorganisms engineered to enhance bioremediation capacity.

### 4.1. Biocontrol Engineered Microorganisms

Biocontrol agents (BCAs) comprise a diverse group of organisms, including bacteria, fungi, protozoa, viruses, insects, mites, and nematodes, that suppress plant pathogens through multiple biological mechanisms [132]. This review focuses on microbial BCAs, particularly bacteria, fungi, and protozoa. Their modes of action include the induction of systemic resistance, production of antibiotics, siderophores, and antimicrobial metabolites such as non-ribosomal peptides (NRPs), ribosomal peptides (RPs), and polyketides (PKs). Among NRPs, cyclic lipopeptides are especially important due to their broad-spectrum activity against fungal and bacterial pathogens. Additional mechanisms include mycoparasitism, competition for nutrients and space, and the production of volatile organic compounds (VOCs) [133,134].

Despite their potential, BCAs do not always provide consistent or effective protection under all conditions. However, recent advances in molecular biology and biotechnology offer new opportunities to enhance their efficacy [135]. Developments in genomics have facilitated the identification, characterization, and improvement of BCAs, including the discovery of biosynthetic gene clusters (BGCs) that underlie their functional traits and mechanisms of action [136]. The effectiveness of BCAs can be further improved through genetic engineering approaches, such as CRISPR/Cas9, gene regulation (over-expression or knockout), and RNA interference (RNAi), among others [137].

For example, Barahona et al. [138] generated a triple mutant of *Pseudomonas fluorescens* F113, targeting the *kinB*, *sadB*, and *wspR* genes (KSW mutant) via directed mutagenesis. This mutant exhibited enhanced motility, increased competitiveness for rhizosphere colonization, and improved biocontrol performance compared to the wild-type strain, demonstrating that enhanced colonization capacity can directly translate into improved biocontrol efficacy. In some cases, beneficial traits from one species can be transferred to another to enhance biocontrol capacity. For instance, Downing and Thomson [139] introduced the *chiA* gene from *Serratia marcescens* into an endophytic *Pseudomonas fluorescens* using a heterologous recombination approach. The resulting strain showed effective biocontrol activity against the phytopathogenic fungus *Rhizoctonia solani* in bean seedlings under controlled conditions.

Another strategy to enhance BCA performance is genome shuffling (GS), which accelerates the generation of genetic diversity and can be effectively combined with random mutagenesis to improve target traits. This approach is particularly suitable for microorganisms with a defined sexual cycle or those amenable to protoplast fusion, allowing iterative recombination among selected parental lines [140]. Through successive rounds of recombination and selection, GS facilitates the accumulation of beneficial genetic variations linked to desirable phenotypes [141].

Originally described by Zhang et al. [142] GS represents a rapid evolutionary strategy that merges features of conventional breeding with DNA shuffling at the whole-genome scale. By enabling multi-parental recombination, it generates highly diverse combinatorial libraries, surpassing the limitations of biparental crosses. When applied to pre-selected microbial populations, GS can yield strains with significantly enhanced performance, including improved metabolite production. A notable example is the increased tylosin yield achieved in *Streptomyces fradiae*, underscoring the value of GS as a non-recombinant tool for microbial improvement and metabolic engineering [143], applied this technique to enhance the biocontrol capacity of *Streptomyces* strains against *Streptomyces scabies* and *Phytophthora infestans*, two major potato pathogens. Six fusants with improved in vitro antagonistic activity were selected. Although all retained the ability to produce geldanamycin, none showed over-production of this antibiotic. Instead, enhanced antagonistic activity was associated with increased diversity of secreted metabolites: while parental strains produced 7–8 metabolites, fusants produced 12–15. Biocontrol assays demonstrated that four of the six fusants provided better protection of potato tubers than the parental strains. These results suggest that improved biocontrol was linked to metabolic diversification rather than over-production of a single compound.

Biocontrol agents exhibit a wide diversity of mechanisms of action, often operating simultaneously or in a context-dependent manner. However, determining which mechanisms are most effective remains challenging due to the high variability in environmental conditions, crop species, and the specific traits of each microorganism, and therefore requires rigorous experimental validation. Relevant examples of genetically engineered microorganisms are summarized in Table 1 and illustrated in Figure 2.

### 4.2. Modified Microorganisms with Improved PGP Traits

#### 4.2.1. Plant Growth-Promoting Bacteria

**Figure 3:**
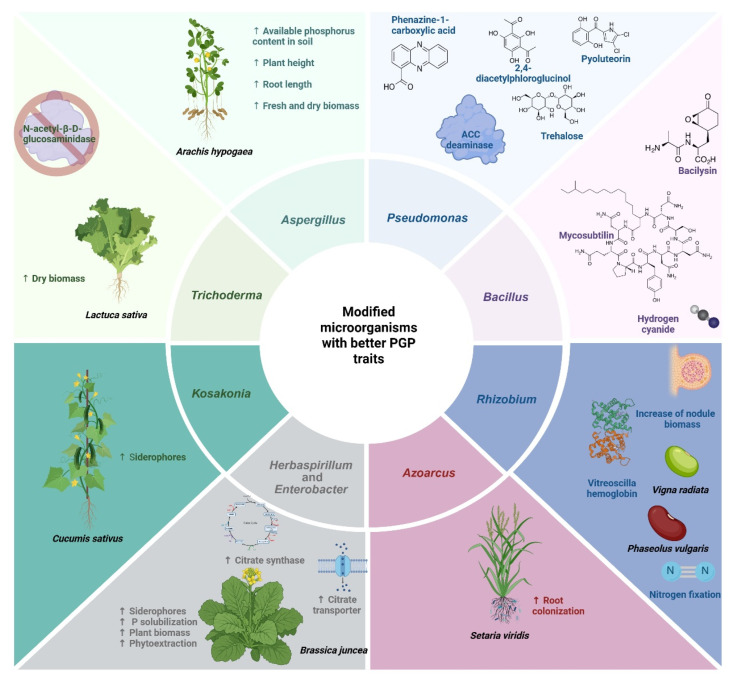
Modified microorganisms with better PGP traits. Overview of genetically modified microorganisms with enhanced plant growth-promoting (PGP) traits and their associated mechanisms. The diagram highlights representative genera featured in this review, such as *Aspergillus*, *Pseudomonas*, *Bacillus*, *Rhizobium*, *Azoarcus*, *Herbaspirillum*, *Enterobacter*, *Kosakonia*, and *Trichoderma*. Created in https://BioRender.com.

Numerous studies have demonstrated the application of both targeted and random mutagenesis, as well as gene over-expression strategies, to enhance key functional traits in plant-associated microorganisms. These traits include root colonization, nutrient solubilization, secondary metabolite production, and tolerance to abiotic stress (Table 2, Figure 3).

Transposon mutagenesis, particularly using Tn5 systems and their derivatives (EZ-Tn5, mini-Tn5), has been widely employed to identify genes involved in essential functions for rhizosphere establishment. For example, in *Kosakonia radicincitans* GXGL-4A, Tn5 mutagenesis enabled the identification of mutants with enhanced siderophore production, which translated into improved cucumber growth [46]. Similarly, in *Rhizobium* sp. MR-54, Tn5 mutagenesis revealed genes associated with increased root nodulation and fructooligosaccharide solubilization, leading to improved performance in mung bean plants [40].

More advanced approaches, such as transposon insertion sequencing (TnSeq), have enabled the systematic evaluation of gene contributions to rhizosphere colonization. In this context, studies on *Azoarcus olearius* DQS4 and *Herbaspirillum seropedicae* SmR1 demonstrated that specific mutations can significantly enhance colonization capacity in *Setaria viridis*, highlighting the value of genetic tools for identifying key determinants in plant–microorganism interactions [57].

In parallel, non-targeted chemical and physical mutagenesis using agents such as N-methyl-N′-nitro-N-nitrosoguanidine (NTG), methyl methanesulfonate (MMS), ethyl methanesulfonate (EMS), and UV radiation has been widely used to generate variants with improved phenotypes. A recent study shows that *Chlamydomonas reinhardtii* (an alga) produces extracellular IAA via LAO1, negatively affecting its growth at high concentrations [152]; however, the plant growth-promoting bacterium *Methylobacterium aquaticum* mitigates these effects while benefiting from the interaction, illustrating a mutualistic auxin-mediated exchange. This highlights the broader significance of IAA as a key signaling molecule in microbe-associated systems, including plant growth-promoting bacteria. Notable examples include strains of *Pseudomonas fluorescens*, *Pseudomonas simiae*, and *Pseudomonas corrugata* with enhanced phosphate solubilization, siderophore production, ACC deaminase activity, and IAA synthesis, leading to improved plant growth and stress tolerance in crops such as wheat, mung bean, and rice [41,44,59]. Similarly, chemical mutagenesis of *Rhizobium tropici* CIAT889 increased nodular biomass in *Phaseolus vulgaris*, further underscoring the relevance of optimizing IAA-associated traits for agricultural performance [39].

Gene over-expression strategies have also proven highly effective in enhancing specific metabolic functions. For instance, over-expression of genes involved in trehalose biosynthesis in *Pseudomonas* sp. UW4 improved plant tolerance to salinity in tomato, underscoring the role of compatible solutes in abiotic stress adaptation [58]. Similarly, over-expression of genes associated with siderophore production and phosphate solubilization in *Enterobacter* sp. NBRI K28 and *Herbaspirillum seropedicae* Z67 resulted in enhanced plant growth and improved nutrient acquisition [45,48].

Furthermore, both classical and modern studies have demonstrated that increased production of antimicrobial metabolites significantly enhances biocontrol activity. Genetically modified *Bacillus subtilis* and *Pseudomonas fluorescens* showed elevated production of compounds such as mycosubtilin, bacilysin, phenazines, and polyketides, leading to improved control of soil-borne pathogens including *Pythium*, *Fusarium*, *Rhizoctonia*, and *Gaeumannomyces* across various crops [49,51,53].

Collectively, these studies demonstrate that genetic engineering strategies have enabled the identification and optimization of key metabolic pathways involved in plant growth promotion and biocontrol. As a result, bacteria are increasingly being developed as versatile and customizable platforms for the design of improved bioinoculants aimed at enhancing crop productivity and health.

**Table 2:** Representative examples of genetically engineered microorganisms for enhanced plant growth.

Modified Microorganism	Genetic Modification Technique Used	Improved Trait	Beneficiated Crop(s)	Reference
*Azoarcus olearius* DQS4 and *Herbaspirillum seropedicae* SmR1	Transposon mutagenesis sequencing (TnSeq) approach	Gene mutations that positively impacted the ability of bacterial strains to colonize roots.	*Setaria viridis*	[57]
*Aspergillus niger v. Tiegh*	Atmospheric Room Temperature Plasma (ARTP)	Improved the P content in the soil. Increased plant height, root length, fresh biomass and dry biomass in pot experiments.	Penut seedlings	[153]
*Bacillus licheniformis* PM7	Ultraviolet radiation, Ethyl methanesulfonate and Ethidium bromide mutagenesis	Increase in phosphate solubilization, siderophore and HCN production, as well as enhanced antifungal activity against *Phytophthora capsici*, *Fusarium oxysporum*, and *Dematophora necatrix*. Additionally, improved root and shoot growth parameters in tomato plants.	Tomato seeds cv. Solan lalima	[43]
*Bacillus subtilis* BBG100	Gene over-expression	Increase in mycosubtilin production and biocontrol of *Pythium aphanidermatum*.	Tomato seedlings	[50]
*Bacillus subtilis* PY79	CRISPR/Cas9 system	Improved Bacilysin production.	-	[49]
*Bacillus subtilis* and *Pseudomonas fluorescens*	UV mutagenesis and Ethyl Methane Sulfonate (EMS) Mutagenesis	Increase in phosphate solubilization, plant height, dry weight, root, shoot dry weight and chlorophyll content.	*Oryza sativa* var. ADT 43	[44]
*Enterobacter* sp. NBRI K28	Gene over-expression	Siderophore over-production and increase in P solubilization, capable of stimulating plant biomass and enhancing phytoextraction of Ni, Zn and Cr.	*Brassica juncea*	[48]
*Herbaspirillum seropedicae* Z67	Gene over-expression	Over-expression of citrate synthase (*gltA1*) citrate transporter (*citC*) genes and increase in phosphate solubilization.	*Oryza sativa*	[45]
*Kosakonia radicincitans* GXGL-4A	Tn5 transposon mutagenesis	Able to synthesize siderophore.	Cucumber seedlings	[46]
*Pseudomonas corrugata* NRRL B-30409	Chemical treatment with N-methyl-N’-nitro-N-nitrosoguanidine	Increase in phosphate solubilization at lower temperatures and growth parameters in wheat.	Wheat plants	[41]
*Pseudomonas fluorescens* ATCC 13525	Chemical treatment with N-methyl-N’-nitro-N-nitrosoguanidine	Siderophore over-production and increase in root elongation at low temperature.	Mung bean	[47]
*Pseudomonas fluorescens* CHA0	Gene over-expression	Over-produces the antimicrobial compounds polyketides 2,4-diacetylphloroglucinol and pyoluteorin and displays enhanced biocontrol activity against *Pythium* ultimum.	*Cucumis sativus* L.	[51]
*Pseudomonas fluorescens* GM BCA	Mini-Tn5 transposon mutagenesis	Constitutive expression of phenazine-1-carboxylic acid and improved biocontrol activity against *Pythium* spp., *Fusarium* spp., *Gaeumannomyces graminis* var. *tritici*, *Phytophtora cinnamomi* and *Rhizoctonia solani*.	Pea, wheat and sugar beet seeds	[53]
*Pseudomonas koreensis* AK-1	Chemical treatment with N-methyl-N’-nitro-N-nitrosoguanidine	Able to induce plant growth in drought stress by PEG. Increase in solubilized inorganic phosphates.	*Glycine max* L. var. JS9560	[42]
*Pseudomonas mosselii* 923	EZ-Tn5 transposome system	Pseudoiodinine over-production and antagonistic activity against *Xanthomonas oryzae* pv. *Oryzae* and and *X. oryzae* pv. *Oryzicola*.	Rice seedlings	[154]
*Pseudomonas putida* WCS358r	Mini-Tn*5 lacZ1* transposon	The antifungal compound phenazine-1-carboxylic acid is constitutively produced and exerts biocontrol activity toward *Gaeumannomyces graminis* var.tritici.	Field-grown wheat	[52]
*Rhizobium* sp. MR-54	Tn5 transposon mutagenesis	Higher root nodulation and P-solubilization.	Green gram	[40]
*Pseudomonas simiae* AU	Chemical treatment with N-methyl-N’-nitro-N-nitrosoguanidine	Increase in ACC deaminase (ACC-D) activity, indole acetic acid (IAA) production and inorganic phosphate (Pi) solubilization. Enhancement of plant growth parameters and drought tolerance.	Mung bean	[59]
*Pseudomonas* sp. UW4	Gene over-expression	Trehalose over-expression and protected tomato plants under salt stress.	*Lycopersicon esculentum* cv. Saladette	[58]
*Pseudomonas putida* WCS358r	Mini-Tn*5 lacZ1* transposon	The antifungal compound phenazine-1-carboxylic acid is constitutively produced and exerts biocontrol activity toward *Gaeumannomyces graminis* var.tritici.	Field-grown wheat	[52]
*Rhizobium etli* CE3	Triparental mating	Expression of *vhb* gene. Bean plants treated with the engineered strain showed increased nitrogenase activity and higher total nitrogen levels.	*Phaseolus vulgaris* cv. Negro Jamapa seeds	[37]
*Rhizobium etli* CFN42	Gene over-expression	Increase in nitrogenase activity, plant weight, and plant nitrogen content. Increase in seed yield, higher nitrogen content, and nitrogen yield in seeds.	*Phaseolus vulgaris*	[38]
*Rhizobium tropici* CIAT889	Treatment with methyl methanesulfonate (MMS)	Increase in nodule biomass per plant.	*Phaseolus vulgaris* L. cv. Carioca BRS Estilo	[39]
*Rhizobium* sp. MR-54	Tn5 transposon mutagenesis	Higher root nodulation and P-solubilization.	Green gram	[40]
*Trichoderma hamatum* GD12	Insertional mutagenesis	N-acetyl-β-d-glucosaminidase gene disruption enhances the growth of lettuce seedlings.	Lettuce seedlings	[55]

#### 4.2.2. Plant-Beneficial Fungi

Similarly to bacteria, fungi—particularly species of *Trichoderma* and *Aspergillus*—have been extensively studied as biocontrol agents and plant growth promoters due to their ability to colonize the rhizosphere, produce hydrolytic enzymes, solubilize nutrients, and suppress phytopathogens (Table 2). In this context, various mutagenesis and genetic engineering strategies have been applied to enhance key functional traits and improve their performance under controlled agricultural conditions.

One of the earliest approaches involved insertional mutagenesis to identify genes associated with fungus–plant interactions. For instance, disruption of the gene encoding N-acetyl-β-D-glucosaminidase in *T. hamatum* GD12 significantly increased lettuce growth. This finding suggests that modifications in chitin-degrading pathways can influence root colonization dynamics and promote plant growth, even without directly enhancing antagonistic activity against pathogens [55].

In addition, non-conventional mutagenesis techniques such as atmospheric room-temperature plasma (ARTP) have been applied to *A. niger* with promising results. This approach induces random mutations that significantly improve nutrient solubilization, particularly phosphorus availability, as well as plant growth parameters including plant height, root length, and biomass in peanut seedlings. These results indicate that ARTP can generate beneficial phenotypic variation without the need for targeted genetic modification [153].

Directed gene over-expression has also been widely used in *Trichoderma* species to enhance biocontrol activity. For example, over-expression of the *prb1* gene in *T. harzianum*, which encodes an extracellular protease involved in the degradation of fungal and nematode structures, led to increased enzymatic activity and improved control of the nematode *Meloidogyne javanica* under infection conditions [56].

Similarly, transformants of *T. harzianum* overexpressing *prb1* showed a significant reduction in disease caused by *Rhizoctonia solani* in in vitro assays. This study also demonstrated a relationship between pathogen suppression and the induction of plant defense-related enzymes, providing a foundation for the development of improved strains with practical agricultural applications [54].

Collectively, these studies demonstrate that both random mutagenesis and targeted genetic modification are effective strategies to enhance fungal traits associated with plant growth promotion and biocontrol. However, most studies have been conducted under controlled conditions, highlighting the need for further evaluation in complex agricultural environments where soil interactions play a critical role.

## 5. Plant-Beneficial Microalgae

One group of PGPM that has not been as extensively explored as bacteria or fungi—organisms that exhibit greater capacity to survive under extreme conditions such as drought—is microalgae. However, their applications in sustainable agriculture and biotechnology are broad (e.g., biostimulants, nutraceuticals, food, aquaculture feed, and biofuels). Some microalgal species belonging to genera such as *Acutodesmus*, *Calothrix*, *Chlamydomonas*, *Chlorella*, *Dunaliella*, *Porphyridium*, *Scenedesmus*, and *Spirulina* have been shown to be excellent producers of metabolites that stimulate plant growth, in addition to enhancing plant immune responses against potential pathogens [155]. Therefore, their cells have been used as bioreactors to produce phytohormones, amino acids, pigments, lipids, and antioxidants, which can be applied as biostimulants in crops [156].

Experimental studies have demonstrated that microalgae are capable of promoting plant growth. For instance, the application of *Acutodesmus dimorphus* has been reported as effective as a biofertilizer and biostimulant in tomato plants [157]. Similarly, extracts of *Chlorella vulgaris*, *Nannochloropsis salina*, and *Arthrospira platensis* applied to bean plants have increased crop yield and improved the nutritional quality of the seeds [158]. Likewise, the application of *Chlorella vulgaris* to seeds and plants of *Triticum aestivum* var. Achtar has been shown to significantly improve wheat germination and growth [159]. Positive effects on seed germination in *Solanum lycopersicum and Hordeum vulgare* have also been documented after the application of *Chlorella vulgaris* and *Scenedesmus obliquus* [160] as well as on the growth of *Beta vulgaris* L. subsp. *cycle*, where *C. vulgaris* S45 showed biostimulatory activity. Together, these studies demonstrate how microalgae can improve plant growth, by producing phytohormones and additional bioactive compounds that play a role in plant growth. In a recent study [161], genomic engineering techniques such as CRISPR/Cas9, TALENs, zinc finger nucleases (ZFNs), and conventional genetic transformation approaches using *Agrobacterium* have been reviewed to enhance the quantity and quality of various bioactive compounds [161]. Microalgae are also being utilized and optimized for bioremediation processes, particularly in aquatic environments. Given the extensive number of high-quality review articles on microalgae and cyanobacteria, as well as their role in biotechnology, including remediation and sustainable agriculture, we recommend the following readings for further insight into the topic [152,161,162].

Despite recent advances in gene-editing tools, the use of genetically modified microalgae to biostimulate plants remains under-researched. Although metabolic pathways have been optimized to generate bioactive compounds, confirmation of these organisms as biostimulants and biofertilizers in plant-microalgae systems remains limited. In this regard, studies demonstrating the potential of genetically modified microalgae to enhance plant growth-promoting traits are presented in Table 3, which summarizes significant research in this developing field.

## 6. Bioremediation of Contaminated Agricultural Soils

Beneficial soil microorganisms, including bacteria and fungi, play a key role in maintaining soil health by participating in nutrient cycling, mineral solubilization, phytohormone production, and pathogen suppression. Many of these microorganisms also possess natural abilities to degrade environmental contaminants. Advances in genetic engineering have enabled the enhancement of these traits by optimizing metabolic pathways, increasing the expression of degradative enzymes, or introducing new catabolic functions. As a result, engineered microorganisms can improve bioremediation efficiency while indirectly promoting plant growth by restoring soil quality and reducing toxicity, contributing to more sustainable agricultural systems.

Soil is a complex and dynamic environment that can be affected by multiple sources of pollution. According to Wołejko et al. [168], five major types of anthropogenic pollutants have been identified.

Among the most persistent contaminants are pesticides, which tend to bioaccumulate and retain their molecular integrity and biological activity for extended periods after their release into the soil. The degradation rates of pesticides tend to vary widely depending on their physicochemical properties and the environmental conditions in which they are found; therefore, their half-life usually ranges from days to months or even years [169].

Another major group of pollutants includes heavy metals (HMs), commonly defined as elements with densities greater than 5 g·cm^−3^. This group comprises metals and metalloids with potential toxic effects, such as arsenic (As), cadmium (Cd), chromium (Cr), lead (Pb), mercury (Hg), copper (Cu), zinc (Zn), and nickel (Ni). In recent decades, HMs have become a significant source of environmental pollution derived from both natural and anthropogenic activities [170]. These elements have been detected at toxic concentrations in soil, water, sediments, and living organisms, posing serious risks to ecosystems and human health [170,171,172]. Phytoremediation has emerged as an important strategy to mitigate soil contamination and can be categorized into phytovolatilization, phytoextraction, phytostabilization, and phytodegradation, depending on environmental conditions and pollutant characteristics [170].

Additionally, the movement of pesticides from soil to water is a significant concern. The transport of pesticides from soil to water is an additional environmental concern. Through leaching processes, pesticides can contaminate groundwater and spread via hydrological systems, extending their persistence and causing both short- and long-term ecological damage. Pesticides may undergo transformation into metabolites through photochemical, chemical, and microbial processes [173]. Microbial biodegradation plays a central role in the detoxification of these compounds by converting complex or toxic molecules into simpler, less harmful forms through specific enzymatic pathways. In this process, microorganisms produce enzymes—such as oxygenases, dehydrogenases, and reductases—that catalyze oxidation–reduction reactions, breaking chemical bonds and transforming pollutants into intermediates that can be further metabolized or mineralized (e.g., to CO_2_ and H_2_O). In addition to direct degradation, complementary mechanisms enhance removal efficiency: biosorption involves the binding of contaminants to cell surfaces; bioaccumulation refers to their uptake and intracellular sequestration; bioaugmentation introduces specialized degraders to accelerate the process; and bioleaching mobilizes metals through microbially driven chemical changes. Together, these processes contribute to the effective remediation of contaminated environments [174].

Key enzymes involved in bioremediation include cytochrome P450 monooxygenases, laccases, dehalogenases, dehydrogenases, hydrolases, proteases, and lipases [175]. Furthermore, advances in genetic engineering have enabled the development of microorganisms with enhanced degradative capabilities for a wide range of pollutants, including synthetic dyes from the textile industry [176], alkanes and aromatic compounds [177,178,179], and pesticides [180,181,182].

## 7. Genetic Engineering of Microorganisms: An Approach to Enhance Heavy Metal Removal and Bioremediation

Microalgae have attracted considerable attention as promising biological systems for heavy metal (HM) removal due to their high capacity for metal uptake and tolerance. Elevated levels of HM resistance have been reported in various phototrophic microorganisms; for example, members of Chlorophyta can tolerate copper concentrations of up to 15 µM [183]. Consequently, microalgae are increasingly recognized as effective and sustainable platforms for bioremediation.

One of the most widely studied models is *Chlamydomonas reinhardtii*, which has been genetically engineered to enhance metal accumulation and tolerance. Over-expression of the metal transporter gene *CrMTP4* in *C. reinhardtii* CC125 improved intracellular metal compartmentalization and tolerance [184]. Similarly, heterologous expression of the plant metal transporter *AtHMA4* increased Cd and Zn accumulation, demonstrating the potential of cross-kingdom gene transfer to enhance metal uptake mechanisms [185]. In addition, chloroplast transformation to express a synthetic gene encoding γ-glutamylcysteine synthetase significantly improved Cd removal efficiency [186]. Likewise, transformation of *C. reinhardtii* using *Agrobacterium tumefaciens* carrying the *ACR3* transporter gene enhanced arsenate uptake from the medium [187].

Genetically engineered bacteria have emerged as powerful bioremediation tools. A well-characterized example is *Synechocystis* sp. PCC 6803, a cyanobacterium engineered to improve Cd and Zn removal. In this strain, the introduction of genes encoding phytochelatin synthases (PCSs) and metallothioneins (MTs) increased intracellular metal sequestration capacity [188]. These cysteine-rich peptides facilitate detoxification through metal chelation and compartmentalization. Furthermore, the expression of *mntH*, *HMP3*, *sodA*, and *sodC* genes enhanced oxidative stress tolerance, potentially improving Pb and Cr removal under metal-induced stress conditions [189].

Yeasts also represent versatile platforms for heavy metal biosorption. In *Saccharomyces cerevisiae* W303-1A, expression of *EC20* significantly improved Pb^2+^ and Cd^2+^ biosorption capacity [190]. Similarly, heterologous expression of the human metallothionein gene *MT2A* enhanced intracellular metal-binding capacity, highlighting the potential of yeast systems for bioremediation applications [191].

Beyond heavy metal removal, metabolic engineering has expanded the environmental applications of genetically modified microorganisms. For example, expression of the enzyme P450 BM3 MT35 conferred the ability to metabolize herbicides in both *Chlamydomonas reinhardtii* and *Bacillus megaterium* TCC 14581 [192]. Likewise, *Escherichia coli* DH5α engineered to express hydrocarbon-degrading genes (*alkB*, *almA*, *xylE*, *ndo*, and *p450cam*) exhibited enhanced degradation of dodecane, benzo(a)pyrene, and crude oil [193]. In addition, heterologous expression of fructose-1,6-bisphosphate aldolase in *Chlorella vulgaris* improved CO_2_ biomitigation efficiency, demonstrating that pathway optimization can enhance both pollutant removal and carbon capture [194].

In the case of molybdenum (Mo), it is an essential micronutrient; however, its excess in irrigation water—associated with mining or geogenic sources—can negatively affect crop growth and metabolism. In this context, algae–bacteria consortia represent a promising alternative for its remediation, as synergistic interactions facilitate metal removal through processes such as biosorption and chemical transformation, thereby improving water quality for agricultural use [195].

**Figure 4:**
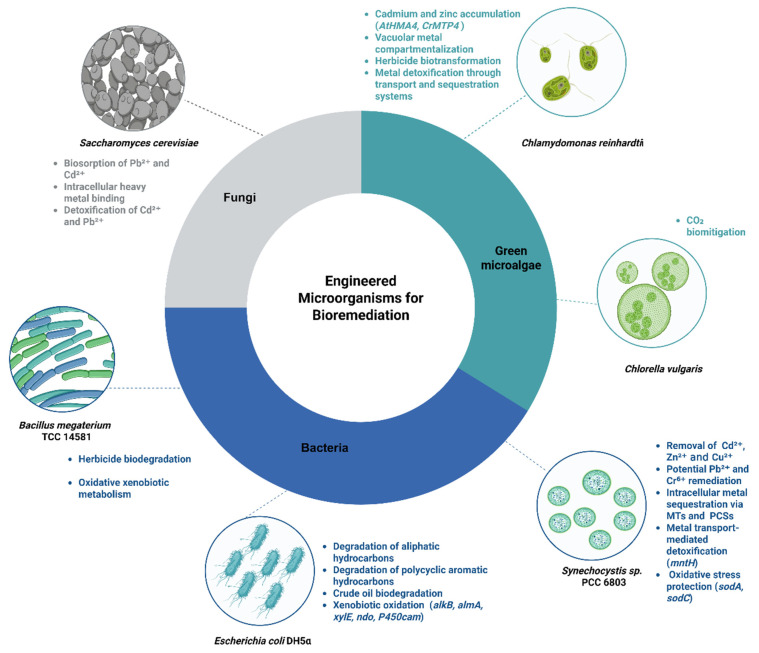
Engineered microorganisms for Bioremediation. Overview of modified microorganisms used in bioremediation applications. The diagram classifies the modified organisms into fungi, bacteria, and green microalgae, highlighting the representative species reviewed in this revision. Created in https://BioRender.com.

Overall, these studies demonstrate that genetic engineering significantly enhances microbial capabilities for heavy metal sequestration, oxidative stress tolerance, and pollutant degradation (Table 4; Figure 4). By integrating metal-binding proteins, transport systems, antioxidant defenses, and engineered metabolic pathways, genetically modified microorganisms represent a promising and scalable strategy for sustainable bioremediation.

## 8. Undesired Results in Genome Engineering: Opportunities for Improvement

When evaluating the potential drawbacks of genetically modified organisms (GMOs), three main aspects are commonly considered: (i) risks to biodiversity and ecosystem dynamics, including impacts on soil, water, and biological communities; (ii) risks associated with gene flow and unintended genetic recombination; and (iii) the potential development of resistance in target organisms [196].

Among these concerns, the loss of biodiversity in non-target organisms remains a major ecological issue [197]. However, evidence regarding the environmental impact of genetically modified crops, particularly those expressing *Bacillus thuringiensis* (Bt) toxins, has been mixed. Bt crops—including cotton, maize, potato, tomato, rice, eggplant, and cruciferous vegetables—have been engineered with genes encoding insecticidal proteins active against larvae of Lepidoptera and Coleoptera. Commonly used genes, including *Cry1Ac*, *Cry1Ab/c*, *Cry1Ac + CpTI*, *Cry1Ac + Cry2Ab*, *Cry1A + Cry1F*, *Cry1F*, *Vip3A*, and *Vip3A + Cry1Ab*, demonstrated no consistent harmful effects of Bt crops on non-target insect populations. A review by Yu et al. [198] reported no consistent harmful effects of Bt crops on non-target insect populations.

Field-based studies further support this perspective. Xing et al. [199] evaluated the impact of Bt maize expressing the *Cry1Ac* protein on non-target arthropods over a three-year period in northern China. Using ecological indices such as species richness, Shannon diversity, and multivariate community analyses, the authors reported the presence of more than 80 arthropod species in both Bt and non-Bt maize systems, with no significant differences in abundance, diversity, or community structure. These findings suggest that Bt maize expressing *Cry1Ac* does not negatively affect non-target arthropod biodiversity under field conditions.

In contrast, more subtle, non-lethal effects have been reported in some cases. Lanzoni et al. [200] investigated the impact of Bt maize expressing the *Cry1Ab* protein on *Rhopalosiphum maidis*, a non-target aphid species. Although no mortality differences were observed, individuals feeding on Bt maize exhibited changes in fecundity, population growth rates, and developmental timing. These results indicate that Bt crops may influence the physiology and population dynamics of non-target organisms, even in the absence of direct toxicity, highlighting the importance of assessing sublethal and long-term ecological effects.

A comprehensive meta-analysis by Meisler et al. [201] provides further insight into these interactions. This study analyzed 233 field experiments conducted between 1997 and 2017 across 13 countries, encompassing 7279 records of invertebrates from multiple taxonomic groups. While Bt maize primarily targets Lepidoptera and Coleoptera, the dataset included a wide range of non-target taxa such as Acarina, Aphelenchida, Araneae, Collembola, Dermaptera, Diplura, Diptera, Haplotaxida, Hemiptera, Isopoda, Lithobiomorpha, Mecoptera, Mononchida, and Neuroptera, many of which play important ecological roles as predators or decomposers. Despite variability among individual studies, the meta-analysis found no consistent evidence of widespread adverse effects of Bt maize on non-target invertebrate communities. These findings emphasize the importance of long-term, large-scale, and comparative studies for accurately assessing the environmental risks associated with genetically modified organisms.

Genetic engineering in microorganisms is often associated with metabolic costs that can compromise cellular fitness and, consequently, functional performance. The introduction and expression of heterologous genes, as well as the rewiring of native regulatory networks, impose an energetic and resource burden on the host, diverting carbon, ATP, and reducing power away from growth and stress tolerance. This trade-off can result in slower growth rates, reduced competitiveness in complex environments such as soil, and decreased persistence under field conditions [202]. Moreover, engineered traits that are advantageous under controlled laboratory settings may become unstable or selectively disadvantageous in natural ecosystems, where microorganisms face fluctuating environmental pressures and competition with native microbiota. As a result, maintaining the balance between enhanced functionality and metabolic efficiency remains a major challenge in the design of robust and effective engineered PGPM for agricultural applications.

## 9. Synthetic Communities

Despite extensive efforts to enhance plant growth-promoting (PGP) activities, the use of single microorganisms often proves insufficient in complex soil ecosystems [203,204]. In addition, individually applied inoculants frequently exhibit low colonization efficiency after introduction into the rhizosphere. This limitation arises because PGP traits are typically the result of interactions among multiple microorganisms. Consequently, the beneficial effects of a single microorganism can be reduced or inhibited by environmental fluctuations, microbial competition, and the lack of synergistic interactions with native microbiota [205,206]. To address these challenges, the reconstruction of microbiomes through synthetic communities (SynComs) has emerged as a promising strategy. SynComs are designed to emulate natural microbial consortia and to modulate specific functions, such as plant growth promotion and stress tolerance [207]. For instance, Aleksieienko et al. [208] developed a SynCom capable of enhancing drought tolerance and reducing wilting and leaf loss in seedlings of *Quercus pubescens* and *Sorbus domestica*.

Similarly, Schmitz et al. [209] designed a SynCom that promoted plant growth in tomato under saline and non-sterile conditions. Their findings highlight the importance of microbial interactions, as synergistic effects among community members can be maintained even in complex, non-sterile environments.

More recently, Liu et al. [205] evaluated a SynCom containing *Rhizobium pusense* TYQ1 as a biocontrol agent against *Meloidogyne incognita*. The SynCom significantly reduced root galls and egg masses, outperforming both individual strains and incomplete consortia, which exhibited lower inhibitory capacity.

In a related study Karanastasi et al. [203], inoculated tomato plants infected with *M. javanica* using a commercial inoculant and two SynComs. All treatments reduced nematode-associated parameters, including egg masses, eggs per root, and total progeny, while also increasing plant biomass despite infection. Notably, SynCom1 showed effects comparable to the commercial inoculant.

Additional studies further support the effectiveness of SynComs under field conditions Fonseca-Garcia et al. [210], developed a SynCom that significantly increased dry biomass in sorghum plants, while Hao et al. [211] demonstrated that a *Trichoderma*-based SynCom enhanced stem length and thickness in cucumber seedlings.

Collectively, these findings demonstrate that SynComs represent stable, functional, and effective microbial consortia for agricultural applications. By leveraging synergistic microbial interactions, they offer a promising strategy for pest control, plant growth promotion, and the development of more sustainable and resilient agricultural systems.

## 10. Future Perspectives

Although genetically modified organisms were initially considered a major innovation in the late 1990s and early 2000s, this approach has significantly evolved. It is now widely recognized that the application of a single microorganism is often insufficient in complex environments, where it must compete with an established and highly dynamic native microbiome. This competitive pressure frequently limits its persistence and functional effectiveness.

In contrast, engineered synthetic communities (SynComs) have emerged as a robust alternative, overcoming many of the limitations associated with single-strain inoculants. SynComs are capable of maintaining functionality under non-sterile, field-relevant conditions and have demonstrated effectiveness in enhancing plant growth and tolerance to abiotic stresses such as drought and salinity. Future strategies are likely to integrate genetically modified bacteria or fungi within SynComs to further improve performance under variable and stressful soil–climatic conditions.

Concurrently, advances in genetic engineering—particularly with the development of precise tools such as CRISPR-Cas—have shifted attention toward the direct improvement of crops with enhanced tolerance to biotic and abiotic stresses [91]. Notable examples include potato and maize varieties with increased drought resistance [92,93,94], as well as *Solanum tuberosum* lines with reduced susceptibility to *Phytophthora infestans* [95] and *Alternaria solani* [212]. Importantly, these strategies are not mutually exclusive; rather, they can be integrated to achieve synergistic effects, provided that environmental safety and ecosystem integrity are carefully considered.

Among the most widely adopted genetically modified crops are those expressing genes from *Bacillus thuringiensis* (Bt) [213]. This bacterium produces insecticidal toxins that effectively control agricultural pests while posing minimal risk to human health. The adoption of Bt crops has led to a reduction in insecticide use [214], along with increased yields and improved economic return [213]. Similarly, virus resistance has been achieved through the introduction of viral genes, reducing susceptibility to infections and enhancing crop productivity [197,215]. In addition, herbicide tolerance—conferred by the insertion of resistance genes—has enabled more efficient weed management, reducing the overall number of herbicides applied in fields with high weed pressure [216,217].

Microalgae present several constraints compared to bacteria when considered as platforms for genetic engineering and field deployment as PGPM. In contrast to many bacterial systems, microalgae generally exhibit slower growth rates, more complex cellular organization, and lower transformation efficiencies, which limit rapid strain optimization and scalability. Genetic manipulation in microalgae often requires more sophisticated tools and selection strategies, and stable expression of transgenes can be affected by gene silencing, positional effects, and regulatory complexity [218]. Moreover, their performance under field conditions is less predictable, as microalgae are highly sensitive to environmental fluctuations such as light intensity, temperature, and water availability, which can impact their survival and functional consistency in the rhizosphere. In addition, large-scale cultivation and formulation of microalgae-based inoculants remain technically and economically more challenging than for bacterial counterparts. These limitations highlight the need for improved genetic tools, robust chassis design, and formulation strategies to fully exploit microalgae as next-generation plant growth-promoting platforms [219].

## 11. Field Performance, Regulation, and Risk Assessment

The increasing demand for food, together with environmental concerns associated with agriculture, underscores the need for more sustainable practices. In this context, GEMs have emerged as valuable tools to enhance plant growth under abiotic stress and phytopathogenic pressure, as well as to improve bioremediation processes. However, their effectiveness under field conditions is often constrained by soil complexity, competition with native microbiota, and challenges related to stability, colonization, and persistence. In addition, plant growth-promoting microorganisms (PGPM) encompass not only bacteria and fungi but also algae, which can contribute to plant productivity through nutrient mobilization and bioactive compound production [220]. Nevertheless, algal performance tends to be highly dependent on specific environmental conditions and may not be consistent across diverse field settings or geographic regions. In contrast, many bacterial and fungal PGPM exhibit greater resilience, partly due to their ability to form resistant structures such as spores, which facilitates their use as inoculants with improved shelf life and survival under storage and adverse conditions such as drought. These constraints collectively highlight the limitations of single-microorganism approaches in replicating the complexity of natural rhizosphere interactions.

The regulatory landscape governing the use of GEMs and other PGPM varies considerably across countries, reflecting differences in risk perception, agricultural priorities, and legal frameworks. In general, regulatory systems aim to ensure environmental and human safety while enabling innovation; however, the level of stringency and the criteria for approval differ widely. For instance, some regions apply precautionary approaches with extensive biosafety evaluations and field trial restrictions, whereas others adopt more flexible frameworks that facilitate commercialization. This heterogeneity can hinder the global deployment of PGPM-based technologies, as products developed and validated in one country may face significant regulatory barriers in another. Moreover, the classification of these microorganisms—particularly when genetic modification is involved—can further complicate approval processes and delay their adoption in agriculture.

Risk assessment is a central component of these regulatory frameworks and typically involves evaluating potential impacts on non-target organisms, soil biodiversity, and ecosystem functions, as well as the likelihood of persistence and horizontal gene transfer. While many PGPM are considered low-risk due to their natural occurrence in soil environments, uncertainties remain regarding their long-term ecological effects, especially under diverse field conditions [221]. Standardized protocols for assessing efficacy and safety are still evolving, and there is a growing need for integrative approaches that combine laboratory, greenhouse, and field data. Strengthening risk assessment strategies, together with harmonizing international regulations, will be essential to ensure the safe and effective implementation of PGPM and GEM-based solutions in sustainable agriculture.

## 12. Conclusions

Genomic engineering should not be applied in isolation but integrated with ecological principles and good agricultural practices, while addressing biosafety considerations, particularly under open conditions. Thus, the future of agricultural biotechnology lies not only in modifying individual organisms but also in designing functional microbial communities. We, therefore, propose the integration of GEMs, SynComs, and multi-omics approaches as they represent a promising path toward more resilient and sustainable agricultural systems.

## Figures and Tables

**Table 1:** Engineering strategies and functional traits of microorganisms used for pathogen control.

Microorganism	Genetic Modification Technique Used	Modified Gene(s)	Target Pathogen or Disease	Benefited Crop(s)	Key Findings	Reference
*Bacillus subtilis* ATCC 6633	Heterologous expression	*spaIFEG* genes	Not evaluated	Not evaluated	Enhanced subtilin production, reaching up to 8.9 ± 1.3 mg/L	[144]
*Bacillus thuringiensis* 3023	Heterologous expression	*chiA* gene	Coleopteran pests	Not evaluated	Significant increase in chitinase activity	[145]
*Clonostachys rosea*	Homologous recombination	*chi67*-*1* gene	*Sclerotinia* *sclerotiorum*	Soybean	Enhanced inhibition of *Sclerotinia sclerotiorum* by up to 81.4%	[146]
*Escherichia coli* HT115 (DE3)	RNA interference (RNAi)	Double-stranded RNAs (dsRNAs) against *AflC* and *BcSAS1*	*Aspergillus* and *Botrytis cinerea*	Maize and *Nicotiana benthamiana*	RNAi-based delivery reduced lesion size by up ~25–35%	[147]
*Pseudomonas fluorescens*	Heterologous expression	*chiA* gene	*Rhizoctonia solani*	Bean	Enhanced antifungal activity	[139]
*Pseudomonas fluorescens* F113rif	Directed mutagenesis	*sadB*, *wspR*, and *kinB* genes	*Phytophthora cactorum*, *Fusarium oxysporum* f. sp. *Radicis-lycopersici*	Strawberry and Tomato	Reduced disease severity in tomato plants	[138]
*Pseudomonas putida* WCS358r	Directed mutagenesis	*phz* or *phl* genes	Soilborne infections	Wheat	Stable colonization in wheat rhizosphere	[148]
*Pseudomonas synxantha* 2-79	Homologous recombination	*prnABCD* genes	*Gaeumannomyces graminis* var. tritici and *Rhizoctonia solani*	Wheat and Canola	Reduced disease severity	[149]
*Streptomyces hygroscopicus* var. geldanus ATCC 55256, *Streptomyces melanosporofaciens* strains EF-76 and FP-54	Genome shuffling	Diversification of secreted metabolites	*Streptomyces* *scabies* and *Phytophthora infestans*	Potato	Increased pathogen inhibition	[143]
*Trichoderma harzianum*	Gene over-expression	The transformants overexpressed the *prb1* gene	*Rhizoctonia solani*	Cotton seedlings	Increased proteinase activity and enhanced biocontrol	[54]
*Trichoderma harzianum*	Gene over-expression	Increase in proteinase Prb1 activity	*Meloidogyne javanica*	Seedlings of tomato cv. 144	Reduced root galling index and increased shoot fresh weight	[56]
*Trichoderma virens*	Homologous recombination	*cht42* gene	*Rhizoctonia solani*	Cotton	Loss of chitinase reduced biocontrol activity	[150]
*Trichoderma virens*	Directed mutagenesis	*tvk1* gene	*Rhizoctonia* *Solani* and *Pythium ultimum*	Cotton	Inactivation of *tvk1* enhanced biocontrol activity	[151]

**Table 3:** Genetically engineered microalgae and cyanobacteria with potential plant growth–promoting traits.

Microorganism	Genetic Modification Technique Used	Gene(s)/Pathway Targeted	Key Finding	Potential Application	Reference
*Anabaena* sp. PCC 7120	Insertional mutagenesis	*hetR*	Disruption of *hetR* abolishes heterocyst formation, confirming its essential role in nitrogen fixation	Target for engineering enhanced nitrogen-fixing cyanobacteria as biofertilizers	[163]
*Chlamydomonas reinhardtii*	Knockout	*LAO1*	Algal IAA production via LAO1 mediates algal bacterial mutualism	Microalgae-based systems to enhance plant growth via auxin production and microbiome recruitment	[152]
*Chlamydomonas reinhardtii*	Promoter engineering	Terpene biosynthesis pathway	Promoter engineering significantly enhances terpene production by optimizing gene expression	Production of bioactive terpenes for plant defense and biostimulation	[164]
*Synechocystis* sp. PCC 6803	Heterologous expression	*nif* gene cluster	Heterologous *nif* expression enables nitrogen fixation in a non-diazotrophic host	Development of nitrogen fixing biofertilizers	[165]
*Synechocystis* sp. PCC 6803	Metabolic engineering and gene over-expression	*aroG*, *tyrA*, *sigE*, and *ppsA*	Metabolic engineering enabled the production of aromatic amino acids and derived phenylpropanoids	Production of plant related bioactive compounds for defense and biostimulation	[166]
*Synechococcus elongatus* PCC 7942	Heterologous expression	*garR*, *mmsB*, *betA*, *msr* and *mcr*	Production of 3-hydroxypropionic acid	Production of organic acids with potential roles in nutrient mobilization and biofertilizer development	[167]

**Table 4:** Examples of genetically engineered microorganisms for bioremediation.

Modified Microorganism	Genetic Modification Technique Used	Improved Trait	Bioremediation	Reference
*Bacillus megaterium* TCC 14581	Homologous recombination	Expression of P450 BM3 MT35 enzyme	Capacity to metabolize Diuron	[192]
*Chlamydomonas reinhardtii*	Heterologous expression	AtHMA4	Accumulation of Cd and Zn	[185]
*Chlamydomonas reinhardtii*	Homologous recombination	Expression of P450 BM3 MT35 enzyme	Capacity to metabolize Diuron	[192]
*Chlamydomonas reinhardtii* CC125	Over-expression	Over-expression of *CrMTP4*	Increased tolerance to Cd	[184]
*Chlorella vulgaris*	Heterologous expression	Expression of fructose 1,6-bisphosphate aldolase	CO_2_ biomitigation	[194]
*Escherichia coli* DH5α	Heterologous expression	*alkB*, *almA*, *xylE*, *ndo and p450cam*	Degradation of dodecane, benzo(a) pyrene and crude oil.	[193]
*Synechocystis* sp. PCC 6803	Heterologous expression	Genes encoding phytochelatins (PCSs) and metallothioneins (MTs)	Remover of heavy metals such as Cd^2+^, Zn^2+^ and Cu^2+^	[188]
*Synechocystis* sp. PCC 6803	Homologous recombination	Expressing exogenous *mntH*, *HMP3*, *sodA* and *sodC genes*	Possible Pb^2+^ and Cr^6+^ remover	[189]
*Saccharomyces cerevisiae*	Heterologous expression	Expression of the human MT2A gene	Biosorption of Cu^2+^	[191]
*Saccharomyces cerevisiae* W303-1A	Homologous recombination	Expression of EC20	Biosorption capability of Pb^+2^ and Cd^+2^	[190]

## Data Availability

Not applicable.

## Abbreviations

The following abbreviations are used in this manuscript:

**ACC deaminase**	1-aminocyclopropane-1-carboxylate deaminase
**ARTP**	Atmospheric and room-temperature plasma
**BCAs**	Biocontrol agents
**BGCs**	Biosynthetic gene clusters
**CO_2_**	Carbon dioxide
**CRISPR**	Clustered Regularly Interspaced Short Palindromic Repeats
**Cas**	CRISPR-associated proteins
**dsRNA**	Double-stranded RNA
**GEMs**	Genetically engineered microorganisms
**GMOs**	Genetically modified organisms
**gRNA**	Guide RNA
**HMs**	Heavy metals
**HDR**	Homology-directed repair
**IAA**	Indole-3-acetic acid
**ISR**	Induced systemic resistance
**NHEJ**	Non-homologous end joining
**NRPs**	Non-ribosomal peptides
**PGPM**	Plant growth-promoting microorganisms
**PKs**	Polyketides
**PAM**	Protospacer adjacent motif
**ROS**	Reactive oxygen species
**RPs**	Ribosomal peptides
**RNAi**	RNA interference
**SDM**	Site-directed mutagenesis
**SynComs**	Synthetic communities
**TnSeq**	Transposon insertion sequencing
**VOCs**	Volatile organic compounds
